# *HEALTH GeoJunction*: place-time-concept browsing of health publications

**DOI:** 10.1186/1476-072X-9-23

**Published:** 2010-05-18

**Authors:** Alan M MacEachren, Michael S Stryker, Ian J Turton, Scott Pezanowski

**Affiliations:** 1 GeoVISTA Center, Department of Geography, The Pennsylvania State University, University Park, PA, USA

## Abstract

**Background:**

The volume of health science publications is escalating rapidly. Thus, keeping up with developments is becoming harder as is the task of finding important cross-domain connections. When geographic location is a relevant component of research reported in publications, these tasks are more difficult because standard search and indexing facilities have limited or no ability to identify geographic foci in documents. This paper introduces *HEALTH GeoJunction*, a web application that supports researchers in the task of quickly finding scientific publications that are relevant geographically and temporally as well as thematically.

**Results:**

*HEALTH GeoJunction *is a geovisual analytics-enabled web application providing: (a) web services using computational reasoning methods to extract place-time-concept information from bibliographic data for documents and (b) visually-enabled place-time-concept query, filtering, and contextualizing tools that apply to both the documents and their extracted content. This paper focuses specifically on strategies for visually-enabled, iterative, facet-like, place-time-concept filtering that allows analysts to quickly drill down to scientific findings of interest in PubMed abstracts and to explore relations among abstracts and extracted concepts in place and time. The approach enables analysts to: find publications without knowing all relevant query parameters, recognize unanticipated geographic relations within and among documents in multiple health domains, identify the thematic emphasis of research targeting particular places, notice changes in concepts over time, and notice changes in places where concepts are emphasized.

**Conclusions:**

PubMed is a database of over 19 million biomedical abstracts and citations maintained by the National Center for Biotechnology Information; achieving quick filtering is an important contribution due to the database size. Including geography in filters is important due to rapidly escalating attention to geographic factors in public health. The implementation of mechanisms for iterative place-time-concept filtering makes it possible to narrow searches efficiently and quickly from thousands of documents to a small subset that meet place-time-concept constraints. Support for a *more-like-this *query creates the potential to identify unexpected connections across diverse areas of research. Multi-view visualization methods support understanding of the place, time, and concept components of document collections and enable comparison of filtered query results to the full set of publications.

## Introduction

Commercial search engines, along with related search methods integrated into information repositories such as PubMed, have revolutionized information retrieval; but, for many real-world challenges they solve only part of the problem. Place and time are fundamentally important to many health science and policy tasks and current search strategies are only just beginning to take them into account. Further, the goal of information retrieval is usually to support some knowledge building or application tasks. Knowledge is often derived by uncovering and contextualizing relations among place, time, and concepts.

The research reported here addresses the challenges of finding relevant information in document repositories and supporting its use through strategies for efficient retrieval, filtering, and contextualization. Visual analytics strategies are introduced that (a) provide quick geographic, temporal, and concept overviews for relatively large initial query results to PubMed (e.g., the 5591 documents retrieved with the query H5N1 OR "avian influenza" OR "avian flu" OR "bird flu"), (b) enable analysts to formulate initial potentially productive queries based on that overview, (c) support query revision and narrowing to find documents of interest quickly, and (d) visually represent some of the place, time, and concept relationships within the focused subset of documents identified. These strategies are implemented in *HEALTH GeoJunction*, an application that provides coordinated computational methods for processing text and visual methods for understanding and interacting with space-time-attribute components of information. Specifically, the application: (a) extracts relevant place, time, and concept information from citations and associated abstracts within large result sets generated from broad queries to PubMed (queries that may generate thousands of returned documents) and merges this information with that derived from MeSH headings, (b) provides dynamically linked maps, time series plots, and concept views for exploring document collections and their properties, (c) supports facet-like drill down to documents that meet specific place, time, and concept constraints, and (d) enables query-by-example capabilities that allow users to quickly find other documents similar to one of interest.

## Background

This section highlights core aspects of the research domains built upon here. We divide discussion into three broad categories of research that we integrate in *HEALTH GeoJunction*: geographic information retrieval; information browsing and filtering; and geo/information visualization and web mapping.

### Geographical information retrieval

Many of the information sources relied upon by health researchers and practitioners contain implicit and/or imprecise geographic data in forms such as postal codes or place names. Finding geographically relevant information, therefore, is more difficult than simply executing a typical web query. It requires application of strategies and methods being developed within the field of Geographical Information Retrieval (GIR). GIR is an active area of research addressing a wide range of questions concerning how to overcome the lack of explicit and precise position information in typical text documents. The primary goals of GIR are to locate documents that are geographically relevant to user queries, to find and interpret geographical references in unstructured text, and to map the document's geographical footprint (the locations for which the document is relevant) as well as the locations of specific entities extracted from the documents [1].

Key steps in GIR, are: (a) place name/term and relation extraction, (b) name/term disambiguation, and (c) geocoding. Extraction involves applying natural language processing methods (sometimes supplemented by machine learning methods) combined with gazetteer look up to identify and extract references to place and (in some cases) relations of place terms with associated entities. A good overview of this process can be found in [2]. Disambiguation includes distinguishing references to place from references to people, businesses, etc. (e.g., Georgia as a person, a U.S. state, or a country; Pennsylvania as a state, part of a university name, part of the name for a utility company, etc.) as well as distinguishing among references to multiple places with the same name (e.g., if the entity is "Springfield," which of the 25 U.S. cities is intended?) [3]. Once the place is determined, the process of geocoding involves determining where places identified are located. This step can be accomplished through methods that range from simple coordinate lookup (e.g., for cities) to complex imputation processes that incorporate multiple supplementary forms of information to precisely locate a specific address [4] or to interpret vague place references (e.g., the Midwest).

Limited attention thus far has been given to the potential role of visualization in support of GIR. Work by Hobona and colleagues, however, proposed a Spatio-Temporal Ontological relevance model that is conceptually related to the place, time, concept filtering approach introduced here. In their system, they demonstrate and assess the potential of sorting retrieval results by locational, temporal, and thematic relevance and propose a 3D visualization method into which these three parameters are mapped to enable visual exploration [5].

One interesting application of GIR methods that is directly relevant to the research presented here is a study by Boulos [6] that explored the geographic characteristics of research contributions to one leading health informatics journal (Medical Informatics & the Internet in Medicine). He used PubMed as the source for publication information and applied MetaCarta http://www.metacarta.com extract geographic information and support mapping of documents. The analysis illustrated the geographic reach of the journal (to 24 countries) and the dominance of European countries as the source of articles (81%).

### Information browsing and filtering

There is a very large literature on information retrieval, document browsing, and interactive query filtering. We highlight a small selection of key ideas from this literature that are built upon in the application presented here. A starting point for thinking about the components of information access to which our GeoJunction application is targeted is Marchionini's [7] categorization of kinds of search into: *look-up *(fact retrieval), *search to learn *(iterative search and filter leading to new or refined knowledge), and *search to investigate *(search that yields research hypotheses, explanations for unexpected results, or cross-connections among potentially related domains that can help derive answers to particularly hard questions). Marchionini groups search to learn and search to investigate into a higher level category of *exploratory search*, the focus of a recent special issue of *Communications of the ACM *[8]. All three search forms are relevant for users of PubMed and related document repositories and all can benefit from support for searching by place and time - the focus of the research reported here.

One particularly successful approach to supporting both fast look-up and exploratory search involves use of faceted hierarchical metadata [9,10]. The core idea involves dividing metadata (data about the data) into *facets *that are usually hierarchically organized; facets are orthogonal categories/dimensions. Using facets, users can quickly narrow their search by making choices to include/exclude all items within a particular facet (or sub-facet). An example relevant to scientific papers in PubMed would be to use the following facets: author affiliations (divided by high-level disciplinary affiliations of medicine, biological science, social science, etc. with each sub-divided into specific disciplines); disease type; screening type; and health care accessibility factors. In this case, a user could quickly select papers by authors from the social sciences, that address a particular cancer, and that consider accessibility to clinics with a particular kind of screening.

Some past research has pointed to the complexity of medical vocabularies as a reason not to apply a faceted approach [11]. On the other hand, other research considers the core medical controlled vocabulary (the Medical Subject Headings - MeSH) to be a faceted thesauri [12]. The difference in perspective seems to be whether facets are considered to be mutually exclusive at all levels; when duplication of an entity in more than one high level category is allowed, MeSH can be considered a faceted classification system. Tang [13] treats MeSH as a faceted classification and reports on a user study that provides evidence for advantages of multi-facet query interfaces (over multiple alternatives) in situations where comprehensive search in both familiar and unfamiliar domains is needed.

The success of faceted systems to support searching and browsing is dependent upon both the choice of facets and the quality of the interface provided to help users leverage the advantage of the approach [14]. The Flamenco system, developed by Hearst and colleagues/students, was designed to test the core concept of hierarchical faceted metadata as the basis for a flexible, visual search and browse interface [9]. Empirical assessment of Flamenco provides evidence that users understand, prefer, and are able to use multiple faceted hierarchies simultaneously to quickly drill down to items of interest [10]. The assessment was carried out with a collection of 35,000 historical art images for which standard metadata facets were supplemented by semi-automated generation of facets produced by relating terms in text descriptions to WordNet concepts.

The core ideas of faceted information classification to support searching and browsing have been adapted to and extended within a range of domains, including: archaeology [15], personal information organization [16], and medical information [13,17]. Most of these efforts have focused on developing the facets [18] and/or automating the process of tagging artefacts so that they can be matched unambiguously to the facets [16]. Beyond the Flamenco project, one of the few efforts to address the user interface challenges of leveraging the potential of faceted classification is FacetMap, an application that applies principles from interactive, information visualization to the task enabling quick, single-click actions to apply facets [19].

### Geo/Information Visualization & Web Mapping

A wide range of interrelated developments in geo/information visualization and web mapping, beyond those represented by FacetMap described above, are relevant to development of *HEALTH GeoJunction*. Here, we highlight three categories, briefly: *tag clouds *for representing concepts; *multi-view, space-time information visualization *for exploring cross-connections in information; and *web map services *for integrating and mapping data from multiple sources.

### Tag clouds

When document (or other artefact) collections are very large, methods are needed to summarize collection content and help users find relevant documents. Many information visualization methods have been developed to support document visualization (e.g., [20-23]). Among the most widely used on the web is the *Tag Cloud. HEALTH GeoJunction *implements and extends this method; thus we focus on it here.

Tag clouds have become popular for content exploration in a range of applications from document collections, through photo repositories, to social networking sites. A tag cloud is a visual information display using words scaled to represent frequency of occurrence in a collection of artefacts. The most common versions of tag clouds display single words arranged alphabetically to support the task of finding terms of interest quickly [24]. Tag clouds can be used to depict user (or computationally) assigned tags applied to any artefact (e.g., photos), formally assigned metadata (as is the case with MeSH headings in PubMed - [25]), or computationally-derived term frequency for all text in a document collection.

In an innovative adaptation of the tag cloud method, Jaffe and colleagues [26] proposed the idea of "tag maps" that plot relevant scaled tags on a map at the geographic locations where they occur. The core idea has been extended in recent work by Wood, et al [27] that integrates tag clouds and tag maps into an application that includes dynamic links between views (see next sub-section for an overview of multi-view visualization environments).

### Multi-view, information visualization

A substantial body of research has focused on developing highly interactive, multiview, exploratory visualization tools that support analysis of space-time-attribute data. With multiview systems, each view enables detailed interactive analysis focused on a component of a complex problem and relations among components are explored through dynamic connections among views (e.g., highlighting a set of points in a scatterplot highlights the geographic locations for those points on a map). The coordinated, multi-view model for visualization applications has been applied frequently to exploratory spatial data analysis tools targeted to health data analysis (e.g., [28-32]) and more recently to web-map applications (e.g., [33,34]).

We build upon and extend this past work here. Most applications of the coordinated multi-view visualization approach (including those cited above), whether or not applied to geographic data, focus on exploring well structured numerical data. Here we extend the approach to support document query, identification of relevant and related documents, filtering documents or concepts by place, time, and related concept, and analyzing the geographic and temporal characteristics of concepts and their relationships extracted from text sources. In addition, most multi-view visualization has been developed for desktop applications. Here we focus on web map applications, thus recent advances in web mapping are also relevant and discussed below briefly.

### Web Mapping

Dramatic advances in web mapping have been achieved over the past several years [35]. Many initial applications were focused on domains such as maps to support tourism [36], planning [37], or deliberation in spatial decision making [38]. Boulos was among the first to explore the potential of web mapping for public health applications. In an early methods paper, he introduced strategies for dynamic database drill-down that link the web map display to an underlying database [39]. He then followed this with a series of reports that detailed ongoing web map advances and demonstrated their relevance for health applications [40-45]. Recent developments have focused attention on support for data-driven exploratory analytical maps that depict aggregate numerical data for census units, counties, etc., (e.g., [33,34,46,47]). In complementary research relevant to the *HEALTH GeoJunction *application introduced here, Boulos [48,49] introduced an approach for semantically-enhanced queries to enable retrieval of information that is conceptually relevant while not necessarily containing the specific keywords used by the information seeker.

As the research and associated technologies outlined above advance, it is becoming possible to integrate them effectively in web services that retrieve, process, and provide flexible access to diverse forms of information linked through common geography. In the public health domain, a project that is directly relevant to the system we introduce here is HealthMap.org http://www.healthmap.org. HealthMap is an automated web service for querying, filtering, integrating and visualizing unstructured reports about disease outbreaks from a range of sources [50]. The system ingests data from many sources including news feeds, World Health Organization announcements, and ProMed mail. The core goals that underlie HealthMap are to classify retrieved reports correctly, provide flexible and useful visualization output, and to scale methods and tools to support increasing volumes of input information and increasing numbers of system users. A particularly innovative part of the HealthMap system is the classification engine that determines, for each document processed, the primary diseases and locations of interest. Accomplishing this requires application of a range of computational, hierarchically applied reasoning strategies that systematically process components of documents to determine both diseases and locations with sufficient precision to be useful and to determine (when multiple locations or diseases are mentioned) which are the focus of attention.

## Results: GeoJunction Design & Implementation

In this section, we introduce *HEALTH GeoJunction*, a highly interactive geovisual analytics-enabled web application designed to assist health researchers, analysts, and policy-makers in finding and contextualizing scientific publications relevant to their needs. A key contribution of *HEALTH GeoJunction *is that *relevance *is addressed systematically and includes geographic and temporal as well as thematic components. More specifically, *HEALTH GeoJunction *implements web services that apply computational reasoning methods to support information extraction tasks; these tasks include determining places documents are about and supporting query-by-example to find documents that are most similar to any determined to be of particular interest. A flexible visual interface with multiple linked views provides users with a combination of cartographic and information visualization methods that support identification of cross-connections among extracted information fragments. Human reasoning is supported through the linked geographic and information visualization displays that enable quick, facet-like, user-defined document partitioning.

**Figure 1:**
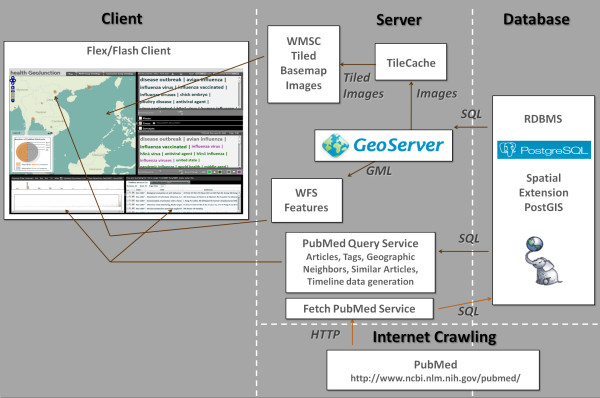
**HEALTH GeoJunction System Architecture**. The primary components in Health GeoJunction are depicted. As indicated, they are organized as a client-server application supported by a spatially-enabled relational database.

The overall *HEALTH GeoJunction *system architecture is detailed in Figure 1. As illustrated, it is a client-server application using open source, geographically enabled database and server technology and our port of OpenLayers to Flex to support mapping in the Flex-based client [51]. *HEALTH GeoJunction *is designed to be generally applicable to a wide range of document repositories. Readers interested in the evolution of our ideas may be interested in brief early discussions (in a poster, abstract, and short workshop paper) of the motivation for the research and illustrations of an earlier implementation that did not incorporate the facet-based query approach presented here [52-54],

In its initial full version presented here, the focus for *HEALTH GeoJunction *is on retrieving and exploring relevant abstracts from PubMed, with relevance including locational and temporal as well as thematic components. The visual interface provides multiple coordinated views organized around an overview map and time series plot summarizing the number of publications produced on a theme by country and over time, respectively. A separate tag cloud view displays the most common PubMed MeSH tags assigned to, and/or terms extracted from, PubMed abstracts.

The remainder of this section provides a detailed (and illustrated) description of the approach taken to the *HEALTH GeoJunction *design and the resulting implementation. First, we present a scenario of *HEALTH GeoJunction *use, to put the discussion of its approach and implementation in context. Next, we describe, briefly, the process of extracting geographic information from documents and geocoding that information (this topic is addressed in more detail in the methods section at the end of the paper). The remainder of the section is focused on design of the visual interface to place-time-concept filtering and exploration and its instantiation in the *HEALTH GeoJunction *application. Subtopics here include development of enhanced tag clouds for exploring concepts in filtered results as they relate to the whole, methods for filtering by place and time, and methods for exploring and contextualizing results.

### A scenario

Scenarios have been demonstrated to play multiple roles in design of information technology [55]. The scenario described here and several others have been used to help develop the functional requirements for *HEALTH GeoJunction *and to iteratively refine functionality as the environment has been developed. Thus, the scenario has provided context for our system development and here it provides context for description of the system. The specific scenario, while hypothetical, is a realistic one and the system supports it fully, as illustrated by example screen shots and an accompanying video http://www.geovista.psu.edu/resources/movies/HealthGeoJunction_2010/HealthGeoJunction_2010.html. The scenario is targeted at sequential, time-place-concept filtering of PubMed abstracts associated with avian flu.

**Figure 2:**
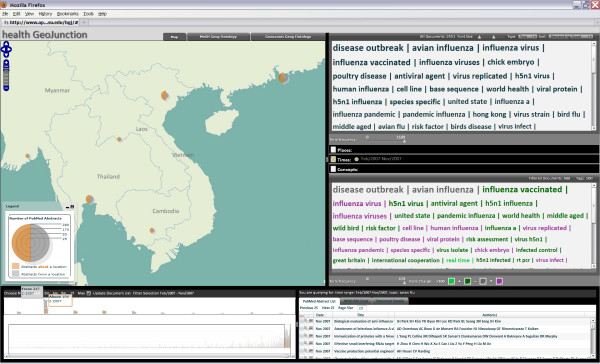
**HEALTH GeoJunction initial view**. This screen capture shows the default view in GeoJunction (after the user has zoomed in on Southeast Asia on the map) for the sample of documents retrieved from PubMed using a query that identified documents about avian flu and related concepts. The map view represents the frequencies of documents that have been determined to be from or about each country with spilt graduated circles (orange/left side representing documents that are about that country or a place within it and gray/right side representing documents from an organization in that country). Below the map is a double timeline, showing a histogram of document frequency for the entire time period represented by documents (below) and for the currently selected time period (above). In this case, the time period selected is February-November, 2007. The tag cloud at the upper right represents term frequency in the full selected data set. The view shows the two-term pair option. The default order is by frequency. Users have the option to select the more common alphabetical order. The lower tag cloud represents the results of current place, time, and concept filtering. Gray terms have not changed their rank order, green terms have higher rank frequency in the selection than in the overall document set (with bright green representing those terms that are not in the top 100 in the full set), and purple represents terms that dropped in rank. The default color choices support users with color vision deficiencies; custom choices can be set by the user. The space between tag clouds represents currently applied place, time, and concept filters. The bottom right tabbed window provides access to a table of documents, pop-up abstracts, geographic footprints (that display on the map), and a browser that shows the document in PubMed.

*An analyst starts with approximately 5000 documents retrieved from PubMed through a query for scientific papers related to avian flu, avian influenza, and H5N1. She obtains a quick overview of main topics in the full document set from the word pair tag cloud at the upper right of the HEALTH GeoJunction interface and is able to compare that to a filtered view in the lower tag cloud (Figure *2 - *the analyst has focused the map on Southeast Asia). The filtered view, by default, shows the most recent documents, in this case the 688 documents for Feb-Nov, 2007. Topics that are more prominent in the recent documents than in the document set overall have terms highlighted in green (with any that were not in the top 100 overall in bright green), those that are less prominent are in purple, and those that are unchanged are in gray*.

*After examining the relative frequency of concepts in the recent documents versus the full set, the analyst starts to consider the place and time components of the information. She looks at the timeline (lower left) that shows the frequency distribution of papers overall in the bottom panel and those within the selected time span as bars in the upper timeline. Geographic characteristics of the document set are shown in the map, using divided graduated circles to depict the frequency of papers that are about places in each country (orange side) compared to papers written by individuals affiliated with institutions in each country (gray side)*.

**Figure 3:**
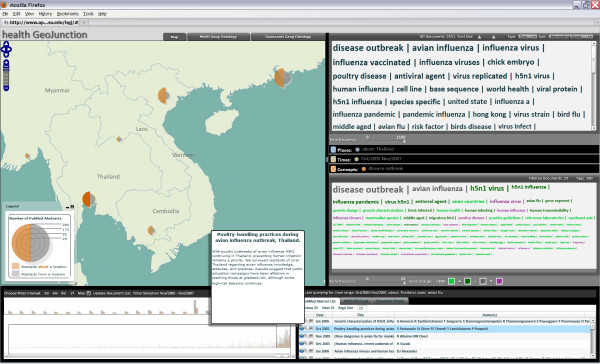
**GeoJunction view with place and concept filtering**. This figure shows the result after the user clicked on the "about" side of the Thailand symbol on the map, filtering the result to only those judged (based on MeSH or GeoJunction feature extraction tools) to be about Thailand, and the user clicked on "disease outbreak" in the top tag cloud to further filter to the subset of documents about Thailand that are also about disease outbreaks. Not surprisingly, H5N1 virus has moved up to be the third most frequent term in this set of documents (now including only 23 of the original set).

*The analyst expands the time focus to the most recent two years, by adjusting the timeline. The updated document set based on the new filter parameters is summarized in the filter tag cloud at the center right (as described above) and papers within the time frame are listed in the lower right panel. The analyst is interested in disease outbreaks (the most common overall term and the 2nd most common in the result set) and particularly those in Thailand. She focuses quickly in on this subset of documents by clicking "disease outbreaks" in the upper tag cloud and the "about" side of the Thailand country symbol on the map. This adds concept and geographic filters to the time filter already in place. That combined set of filters is reflected in both the lower tag cloud and the document list that has now been narrowed to 23 documents (Figure *3*)*.

**Figure 4:**
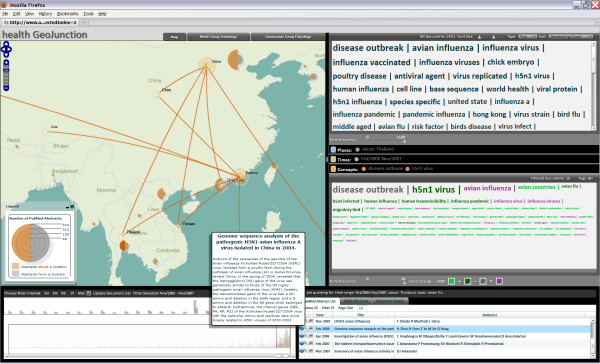
**Geographic footprints**. This figure represents subsequent exploration in which the user has identified two papers of interest. The geographic footprints of both are depicted on the map and the abstract for one is highlighted.

*Finally, the analyst adds one more filter (h5n1 virus), narrowing to 10 documents and scans the document list to find two papers of interest 'Establishment of multiple sublineages of H5N1 influenza virus in Asia: implications for pandemic control' and 'Genome-sequence analysis of the pathogenic H5N1 avian influenza A virus isolated in China in 2004.' She mouses over the "abstract" button of the first paper to get a quick look at the abstract and then clicks the "places" button to generate a map view. Then, she does the same for the second paper (Figure *4*). The map shows the document sources (Hong Kong and Hubei, China) and the countries that the papers focus attention on (primarily in Southeast Asia). Both papers share a geographic focus on China, Thailand, and Vietnam, with each including references to some additional places. Based on the abstracts, she decides that the first paper is particularly relevant and clicks the More-Like-This button to retrieve other closely related documents from the repository*.

Parts of the scenario above could be supported by current PubMed search capabilities. The following query to PubMed retrieves 36 documents that include those of interest in the scenario: *("2005/10" [Publication Date]: "2007/11" [Publication Date]) AND "disease outbreaks" AND H5N1 AND Thailand*. But, to issue this query, the analyst would need to know what time range might include relevant papers. Also, if they were not already interested in "disease outbreaks" there is nothing in PubMed to tell them that it is the most common concept in the set of all avian flu related documents. Similarly, if analysts did not already know that Thailand had several publications about disease outbreaks within the time window of interest, they might not even try Thailand as a place of interest. Further, if they did not know that "disease outbreaks" was a MeSH term and typed "disease outbreak" instead, they would miss the interesting papers entirely (since the papers do not include the term "disease outbreak" and PubMed does not recognize close matches to MeSH terms).

In addition to the above information foraging enhancements, PubMed currently provides no visual (or other) depiction to help analysts understand the possible relationships among papers of interest (e.g., that pairs of papers have multiple countries in common, not just the country used in the query). Further, it includes no "more-like-this" follow up query capability and no easy way to issue a query for papers that are about a place plus its neighbours (for the scenario, about Thailand or any one or more of Laos, Cambodia, or Myanmar); both are included in *HEALTH GeoJunction*. The latter *place + neighbours *query is implemented through a simple Ctrl-click on the "about" symbol for any country of interest, without any need for the analyst to know the names of all neighbouring countries.

### Extracting information from and geocoding documents

To support the use scenario above and related functionality, *HEALTH GeoJunction *takes advantage of existing tags and related metadata (e.g., MeSH headings), extracts concepts and places from text, and integrates the two. The places are geocoded to plot on the map and to support queries for places 'near' a named place. When applied directly to documents in a repository (e.g., abstracts in PubMed), *HEALTH GeoJunction *document processing includes three steps: (a) extract place names and concepts, (b) disambiguate/geolocate places names, and (c) geocode the result and encode it in a spatial database. Each is described briefly here and in detail in the Methodology section.

#### Extraction

When targeted at PubMed, *HEALTH GeoJunction *applies computational methods to extract three kinds of information: places specified in author affiliations, places that documents are about (if mentioned in the title or abstract), and concepts that documents are about (if mentioned in the title or abstract). Information extracted is merged with complementary topic and geographic information encoded in MeSH headings.

#### Disambiguate/geolocate

In general, affiliations listed in PubMed records are precise enough that disambiguation problems occur only rarely. But, places extracted from abstracts are frequently ambiguous, thus computational methods have been implemented to make reasoned choices among alternative places that a label may refer to; improving disambiguation methods is an ongoing research focus, the current approach is described in the methods section below.

#### Geocode

The process of disambiguation involves comparing possible place names against the GeoNames database. GeoNames currently includes more than 7 million unique features including 2.6 million place names (and 2.8 million alternate names). Once a likely match is made, geographic coordinates are retrieved; while GeoNames can provide matches and coordinates for regions and cities within countries, our current implementation aggregates up to countries so that frequency counts by country can be made (for both documents from and about each country). This is needed to support practical place-based facets.

### Visual interface to place-time-concept filtering and exploration

Details for each document retrieved from PubMed are encoded in the PostgreSQL database that supports the system. The web client side of *HEALTH GeoJunction *provides a flexible visual interface to tools that support place-time-concept queries, filtering of queries, and contextualization of results; tools apply to both the documents and their extracted contents. Most existing facet-based query/browsing interfaces rely upon pre-created facets designed to support typical user queries efficiently. In *HEALTH GeoJunction*, we implement a facet-like approach that allows users to apply more control, on-the-fly as they work. The primary, high-level facets are place, time, and concept. These three core facets are presented to the user though the map, timeline, and tag clouds respectively.

This section details the key characteristics of the *HEALTH GeoJunction *visual interface. It starts with a discussion of the flexible facet-like browsing capabilities implemented. Then, we present the extended, overview+detail version of tag clouds developed for and implemented within the application. Then, the methods and tools for place-time query and contextualization are outlined. Finally, the query-by-example capabilities are described.

### Exploring concepts through enhanced tag clouds

*HEALTH GeoJunction *uses tag clouds to depict document term frequency in overview and detail views. Generating a tag cloud from our PostgreSQL database is relatively easy due to the pre-processing done as documents retrieved from PubMed are entered into the database. The server simply makes the required query and generates a result set with the common terms in the specified documents and the term frequency. The web client styles the words so that their size varies in relation to their count. By constructing links that execute new queries when a user clicks on screen elements (e.g., the words in the tag cloud) we create an interface where a naïve user can construct complex SQL queries simply by following a series of links on words or concepts that interest them.

The overview tag cloud in *HEALTH GeoJunction *depicts the frequent terms for the entire repository (Figure 1 - top right) while the detail view depicts the result of the combined place-time-concept filtering applied by the user (Figure 1 - middle right). Tag clouds show terms in order of term frequency by default so that the user is always able to explore key concepts in a subset of documents as they relate to the context of the entire repository. The system also allows users to switch to standard alphabetical order. Terms in the result tag cloud are colour coded to indicate whether they are ranked higher, lower, or the same as in the overall document set. Additional controls are provided to highlight only the most frequent terms (by adjusting a frequency slider) and to adjust the font scaling to fit more or fewer terms into the tag cloud. The latter is useful to support both individual work at normal reading distance and collaboration of 2-3 people around a screen or generation of views that are legible for a presentation.

In the initial version of the application introduced here, users have the option of four kinds of tag cloud: (1) single term tags that are computationally extracted from abstracts by *HEALTH GeoJunction *tools described above, (2) two-term tags that are also extracted by *HEALTH GeoJunction*, (3) tags generated using the Yahoo! Term Extraction Web Service that takes a piece of text and returns a list of significant phrases (see: http://developer.yahoo.com/search/content/V1/termExtraction.html), and (4) MeSH headings that are included in the document metadata from PubMed. The GeoJunction-produced, two-term tags are set as the default. We have found them to be particularly useful, since term pairs often have the necessary qualifiers to sort out specific concepts from general ones (e.g., avian influence versus avian influenza, wild bird versus bird flu, world health versus health policy, risk factor versus either high risk or factor scores, etc.). Two-term tag clouds combined with the iterative ability to add multiple tags to a document filter provide an alternative to computational relation-based document retrieval (for the latter, see: [56]).

### Filtering by time and place

Users are provided with multiple representations and forms of interaction to control both the temporal and geographic filtering and to understand the place and time context of resulting documents. These are discussed below.

#### Time

When the user starts *HEALTH GeoJunction*, a default temporal filter focusing on the most recent publications is applied. This temporal filter gives users a quick overview of any recent changes in emphasis for publications, as reflected by the filtered tag cloud that signifies changes in rank order of concepts in relation to the overall document set.

The timeline view controls the time facet, through which users can apply temporal filtering (Figure 1 - lower left). The lower portion represents the full data set with a histogram that shows monthly frequency of documents having *from *(gray bars) and *about *(orange bars) tags. A double ended slider allows the start and end point to be set for the time filter and users can drag the range along the timeline (e.g., to explore two-year patterns over time). Above the timeline that supports filtering is a second temporal histogram representing the currently selected time window. A mouse-over allows users to determine the number of documents with from and about tags each month. Users can also click on options to specify a fixed temporal window (of 3 months, 6 months, 9 months, or 1 year) or expand to the full time (thus removing the temporal filter).

#### Place

The map view complements the time view by controlling the place facet through which users can apply locational filtering. Users can apply place filters directly through the map or through a geographic location tree view designed to make application of complex place filters easier. Through the map, users apply filters by clicking on place symbols. All papers that are *about *a country (thus that discuss the country or a location within it) can be selected by simply clicking on the 'about' side of the map's graduated symbol depicting the number of publications. Similarly, clicking on the 'from' side of the symbol selects all papers for which the first author affiliation is in the chosen country.

Two forms of more complex query filter can be generated through map interaction. First, a filter that finds all papers that are about (or that are from) an arbitrary set of specific countries can be generated by *Shift_clicking *on any multiple countries and then on "apply filter". This generates an OR filter, returning any papers that are about (or from) any of the selected countries. Alternatively, a filter for papers about (or from) any single country OR any of its immediate neighbours can be generated by a *SHIFT_click* on the symbol for the focus country.

Two geographic tree views provide additional functionality and are designed to facilitate complex queries. The GeoNames view depicts, as a hierarchical tree, all countries in the world, grouped by continent. When users select places in this tree, the filter selects all papers that are about or from the specified countries as determined by either the *HEALTH GeoJunction *entity extraction or by MeSH headings for the publications. The user also can choose to apply an AND query rather than the default OR query (using a radio button choice) - thus making it possible, for example, to find only those publications that mention both Vietnam and Thailand. The MeSH tree provides only those geographic places that are currently part of the MeSH term set; this is a subset of places included in GeoNames. The MeSH tree functions in the same way as the GeoNames tree, but it applies the filter only to MeSH terms attached to publications, thus restricting the result set to those papers that have been hand coded as being about (not from) the place.

### Exploring and contextualizing results

**Figure 5:**
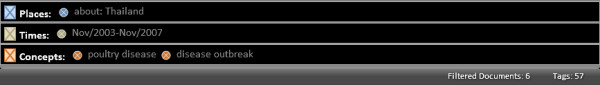
**Place, time, concept filter control**. This figure shows a detailed view of the facet-based filtering control in which users see the place, time, and concept filters that they have applied.  Users are able to selectively remove any of those filters.

As the user applies filters and sees results, they can easily add or remove whole facets (place, time, or concept) or turn off specific choices made within a facet (Figure 5). The facet view, between the tag clouds, shows the user the set of filters currently applied and allows users to quickly remove selective filters from the list or to remove all place or all concept filters at once. Once a user has narrowed in on a set of documents that are likely to be relevant, based on the place, time, and concept filters they have applied, they can explore the documents in more detail using several additional tools.

Document information is provided in the lower right window. Publications meeting the filter criteria are listed here (by default, in sets of 25). Abstracts can be accessed by mouse-over. Clicking on a title generates a query to PubMed with the resulting html page returned by PubMed displayed in a tabbed view. Users can also generate the "geographic footprint" of the publication through the 'Place' button for any paper that has been determined to have a place focus (indicated by tiny globes in the document list). The footprint represents the publication's 'from' location with a multi-pointed star and one or more 'about' locations with a concentric circle symbol connected to the source by leader lines. The place information for multiple papers can be merged on the map through successive clicks (as illustrated in Figure 4).

A final capability implemented in *HEALTH GeoJunction *is support for query-by-example (a more-like-this query). Specifically, users can pick any publication they are interested in and select a 'click for similar articles' icon to retrieve a rank ordered set of documents similar to the one of interest. To support this capability, we make use of the Lucene *MoreLikeThis *query which calculates the sum of the term frequency (tf) and the inverse document frequency (idf) of terms in the original document. The term frequency (tf) is the number of times a word (or term) occurs in a document. For computational efficiency Lucene only considers words which occur more than 2 or 3 times in the original document. By multiplying the term frequency by the inverse document frequency (how many documents are in the index divided by how many contain the term) the algorithm can exclude common words like "the" and favour uncommon words which are of more interest to the user. Once the most "interesting" terms have been determined a new search is carried out for documents that contain all or some of those terms. Currently document similarity is based on the title, abstract, and an OR query of the MeSH terms for the article. The query is executed with a SHOULD match as opposed to a MUST match. As described above, a collection of words which "identify" the example document are used in the query. In a MUST match, all of the identifying terms must occur in a document to be considered similar. A SHOULD query only requires that some of the terms match, although the more that match the more similar a document is considered to be.

The Lucene-based more-like-this approach produces reasonable results (based on informal review) but there are a range of alternative methods to consider. We have designed the tools to make it possible to integrate and compare multiple methods for finding related documents. Among those we intend to assess are Kohonen-style self-organizing maps and pathfinder networks, both of which have been applied with promising results by White, and colleagues [57].

The overall functionality of the system is summarized in Table 1 below.

**Table 1:** Visual interactions to support analyst tasks by facet dimension.

Dimension	Task	Visual Interaction
Time	Filter documents by time range	Size and drag date range slider widgets in timeline

Time	Identify article count by month	Mouse-over time line bar chart

Time	Filter documents by predefined time interval	Click corresponding interval button above timeline and then place time slider

Concept	Filter documents by keyword from corpus	Click tagcloud keyword

Concept	Filter documents by keyword in selected set	Click 'filtered' tagcloud keyword

Concept	Compare frequency of keyword in corpus versus selected set	Click color assignment widget to classify keyword frequency change by color

Concept	Highlight tag frequency above document count threshold	Dynamically highlight tags in tag cloud for full corpus or current selection using respective slider

Concept	Retrieve documents by alternative tagging scheme	Select tag format for tag clouds from drop-down list

Concept	Retrieve documents by low frequency tag	Select sort tag cloud by count from drop-down list, scroll and click tags

Concept	Retrieve documents by specific keyword tags	Select sort tag cloud alphabetically from drop-down list, scroll and click tags

Place	Select documents about a country	Click 'about' country graduated symbol

Place	Select documents from a country	Click 'from' country graduated symbol

Place	Select documents about a country or adjacent countries	Shift-click a country graduated symbol

Place	Select documents by place within geographic hierarchy	Tab from map to geographic tree view and click place names

Place	Retrieve summary of documents by country ('about' or 'from') and time range	Mouse-over country graduated symbol on map

Place	Inspect geographic distribution of documents for query parameters	Map supports standard pan by dragging, map scale slider and buttons for pan by fixed interval

Results	Preview document in result set	Mouse-over icon in result set table

Results	View map representation of places referenced in document	Select globe icon in result set table

Results	Retrieve similar documents	Click 'more-like-this' icon in result set table

Results	Inspect retrieved document	Select row in result set table and then document details tab

All	Remove place or tag constraint	Clear to remove facet-like stub for criterion

## Discussion

There has been limited attention to leveraging the wealth of potentially relevant geographic information contained in research publications and other documents. While inclusion of geography in MeSH headings is a start, it is not sufficient to support effective geographically-constrained filtering and contextualization of publications. At the same time, substantial advances have been made in visually-enabled information foraging, but there has been limited impact of that work beyond domains such as genetic research.

There are many potential benefits to be derived from the development of visual interfaces to support document foraging and tools to extract and utilize geographic references from text documents. With visual interfaces, the immediate feedback and multifaceted contextualization of documents and their cross-connections can help a researcher find relevant documents quickly as well as recognize unexpected connections among research domains. Some of these connections are likely to be grounded in place (e.g., a study of health of workers in the poultry industry focused on practices in a particular country or region may prove to be relevant to subsequent research on human incidence of H1N1 in the same region).

As outlined above, our initial *HEALTH GeoJunction *application focuses on country-level geography since that is the practical level for facet-based filtering. But, once papers within a country or region are found, there are situations, such as the poultry example above, where an analyst may need to drill-down to explore particular cities and towns mentioned. Computationally, our current system can support identification of this finer geographic detail. We plan further interface design/visualization research to extend our overview+detail strategy to make this information accessible and usable.

The conceptual design for the application presented is easily extensible to other applications beyond searching PubMed. Everything presented has been implemented to meet Open Geospatial Consortium (OGC) standards. The database and server used are open source, OGC compliant applications. Thus, other developers can easily take advantage of ideas presented here. The client interface has been implemented using Adobe Flex (a commercial product), but the Action-Script code we developed will be open source. The mapping part of the application is developed on top of our FlexLayers web mapping software (a port of Open Layers). It is available as open source software (LGPL license) at: http://code.google.com/p/flexlayers/. FlexLayers has been used as the basis for, and extended considerably by, the OpenScales project http://openscales.org.

## Conclusions

Given the increasing attention to *place *in public health research, (e.g., due to identification of geographic variation in incidence of autism [58]; concerns about expanding territory of vector borne diseases such as malaria and chikungunya as a result of environmental change combined with increased resistance of disease to antibiotics [59]; and many others), the ability to quickly forage through large repositories of scientific documents using place as a key filter is critical. The initial *HEALTH GeoJunction *application provides a proof-of-concept demonstration that the strategy of place-time-concept document filtering using a facet-like approach is practical and potentially useful for scientific document foraging. For example, the current implementation makes it possible, with 3 mouse clicks, to locate the 15 documents (from a set of approximately 5000) that are about avian influenza and disease outbreaks in Indonesia and published in 2006. Then, one mouse drag can show documents about the same topics and place that were published a year later.

Use of the current system to carry out document filtering tasks such as that described above and in the earlier scenario section can be viewed in the video cited there or can be tried with the live system on the GeoVISTA Center web site: http://www.apps.geovista.psu.edu/hgj. To provide a more complete assessment of the approach and its implementation, our future research will include a multi-step distributed usability assessment following the approach we developed in our previous work to assess and refine our online cancer atlas [34].

The *HEALTH GeoJunction *prototype introduced here has several limitations that we are currently working on. First, it requires an external query to PubMed and a manual step of entering the result into our database. We have developed the methods to read PubMed RSS feeds on a daily basis and add to the original set over time. Our next step is to implement methods that allow users to launch a PubMed query from GeoJunction and have results automatically loaded into the database for subsequent filtering. Second, performance of the current system needs to be improved. While our server-side document processing functions work in real-time (sub-second response) for data documents sets in the 6,000 to 10,000 range, the overhead of the flexible facet-like interface slows response to a range from seconds to tens of seconds. We are currently re-engineering the client application to eliminate this bottleneck. As part of that re-engineering, we will add in a user option to apply the MetaCarta geographic name extraction methods. This will allow users to compare results and pick the method that they feel is the best match to their needs. It will also enable us to do a direct performance comparison of the recognition, recall, and processing speed for our approach (that combines the OpenCalais web service to extract place names and our own algorithm to improve disambiguation) with MetaCarta's results.

PubMed is obviously not the only source of documents that are relevant to public health and that contain geographic references. In complementary work, we are developing methods to retrieve geographically relevant documents from news media and other sources and have applied the tools to forage for insights on human population migrations that are relevant to measles epidemics in Niger (Tomaszewski, Blanford, Ross, Pezanowski, and MacEachren: Supporting Rapid Sense Making in Diverse Web Document Foraging, submitted).

The approach to document filtering and exploration presented, is also not limited to health documents. The current implementation is tuned to work with PubMed abstracts and to take advantage of information contained in MeSH headings. But, the overall facet-based, time-place-concept document filtering approach is a generic one that is potentially applicable to any documents. Of course, for documents that do not contain an explicit distinction between the location a document is from or about, there is an added challenge to distinguish among those two kinds of place. Our current implementation does not include capabilities to do that, but we believe the current implementation is easily generalizable to any formal publications that cite the affiliation of authors and mention places in the content. We are currently assessing this contention with an extension of the system to support filtering and exploration of grant abstracts from the U.S. National Science Foundation.

A future research goal is to combine these efforts to develop geovisual analytics support for an information foraging and sensemaking process with tools to integrate heterogeneous information in support of public health research and practice.

## Methods: GeoJunction Server Implementation

**Figure 6:**
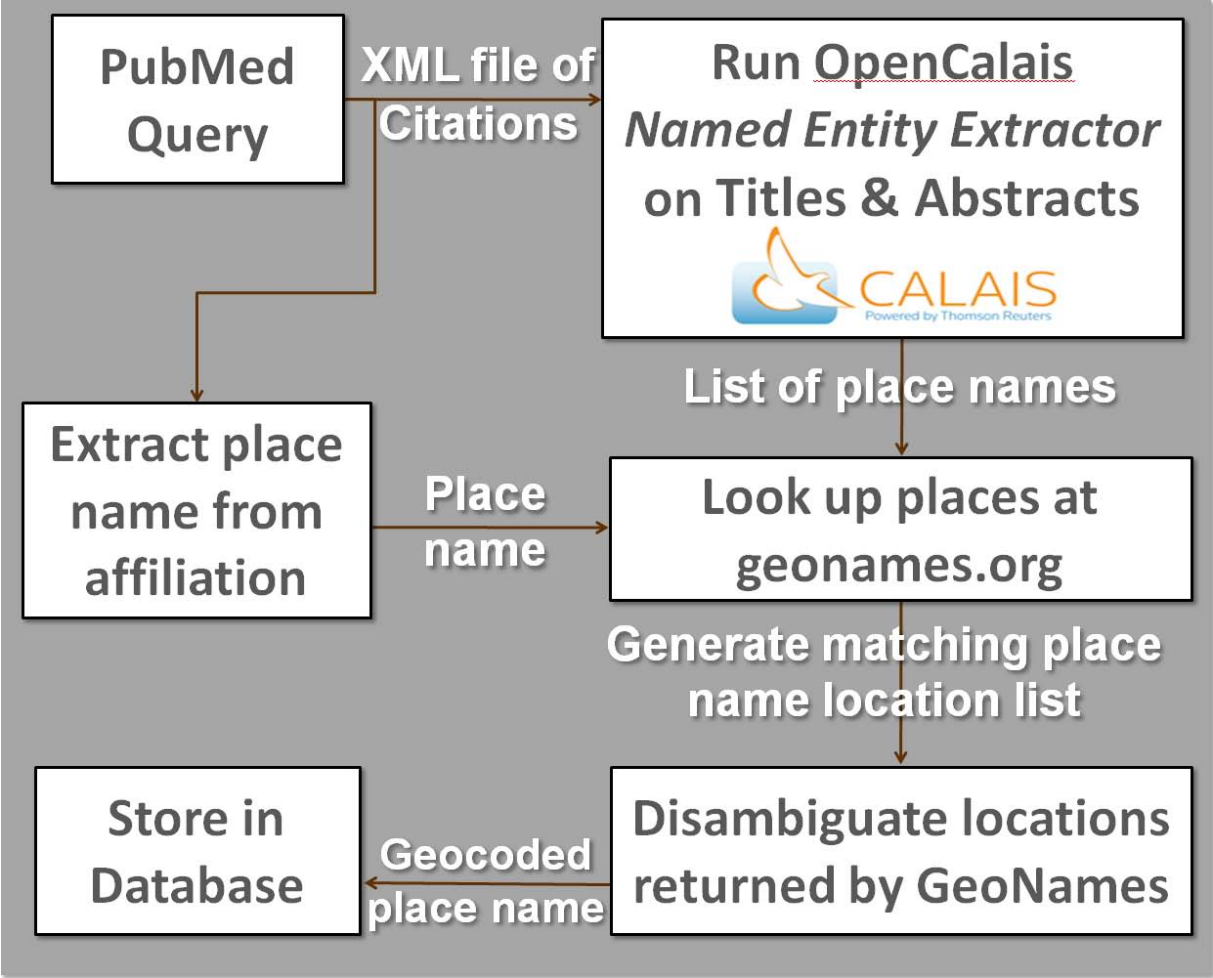
**Document processing components**. The component steps in extracting and geocoding geographic information found in MeSH headings as well as in the title and abstract are delineated here. The approach relies on the OpenCalais named entity extractor to identify geographic references in free text and on the GeoNames database of place names in the world to find geographic entity matches.

In this section, we provide additional details about the server-side components of *HEALTH GeoJunction*. The components were introduced in the Results section and are described schematically in Figure 6. In subsections below, we outline methods for achieving three core steps in the process: (1) extracting place names and concepts, (2) disambiguating/geolocating place names, and (3) geocoding the result.

### Extracting place and concept information

When targeted at PubMed, *HEALTH GeoJunction *applies methods that extract three kinds of information: places specified in author affiliations, places that documents are about (if mentioned in the title or abstract), and concepts that documents are about (if mentioned in the title or abstract).

The easiest of these elements to extract is the place of author affiliation. In the PubMed database these data are returned as a separate field (though only for the first author). In general, authors write their address in a standard way, conventionally with the largest area to the right and smallest area to the left. Thus a simple algorithm splits up the affiliation string, read from the database at each comma to give a series of tokens (in language processing, text is typically segmented into linguistic units such as words, punctuation, numbers, or alphanumeric characters; the units are known as tokens). Tokens are then processed in a right to left order, if commas are missing or fail to give a result the phrase is split at spaces.

The first step is to scan the tokens for a country name or abbreviation; this is done using a simple dictionary lookup. Next, the tokens are scanned for a zip or postal code. We use GeoNames, a web service for geocoding [60], to convert the information to known places. GeoNames provides locations to match the postal codes for more than 35 countries; in some cases a postal code is represented by a regular expression of letters and digits (for example in the UK and Canada) while in other countries a string of digits is used (e.g. USA, France). The system scans the list of tokens and extracts any string that may be a postal code and performs a look up for that code to get a location from the GeoNames service. If the system is unable to discover a postal code then it proceeds to process tokens to the left of the country name (or abbreviation) discovered in the first step (or continues processing tokens starting at the far right if no country was detected). Finally, the GeoNames service is called to look up places with a name that matches the remaining tokens. This look up is currently constrained to the country (if found), but the system can easily support lower level geography. When more than one place is found our system uses a disambiguation algorithm based on the feature code of the locations (as shown below).

### Disambiguation Algorithm

Given two locations A and B:

• Choose A if A is a Political Entity and B is not,

• Choose B if B is a Political Entity and A is not,

• Choose A if A is a Region and B is not,

• Choose B if B is a Region and A is not,

• Choose A if A is an Ocean and B is not,

• Choose B if B is an Ocean and A is not,

• Choose A if A is a Populated Place and B is not,

• Choose B if B is a Populated Place and A is not,

• Choose A if A's population is greater than B's,

• Choose B if B's population is greater than A's,

• Choose A if A is an Administrative Area and B is not,

• Choose B if B is an Administrative Area and A is not,

• Choose A if A is a Water Feature and B is not,

• Choose B if B is a Water Feature and A is not,

• Choose A.

Turton [61] discusses in greater depth the tests applied to the disambiguation algorithm implemented here. Briefly, the previously reported initial results for the disambiguation algorithm showed that the system was approximately 70% successful in correctly choosing the right location for a text string. Subsequently, fixes to the algorithm to handle the problems identified by Turton [61], as implemented in the current *HEALTH GeoJunction *application reported here, have led to an increase in accuracy to approximately 85%.

Tokens are processed until the smallest geographic entity identifiable from the GeoNames service is determined. If the location returned after processing the first token is an administrative area, region or other large area, the algorithm stores this location and continues processing. If the location is a populated place (thus a city or town), processing halts since GeoNames does not provide locations below the populated place level. If processing continues, the location that was stored will be replaced if a "better" location is found by processing tokens to the left of the string.

Once author affiliation location is geocoded, the system moves on to the task of processing abstracts to extract places and other entities mentioned in the text. This is a harder process since the program must first extract the entities from 'raw' text. For place entities, general references to places in the text of an abstract or paper are much less detailed than an affiliation string in a paper (e.g., postal codes are seldom mentioned).

The extraction of named entities is a complex subject in its own right and draws on open source tools to accomplish it. We make use of OpenCalais, a web service provided by Reuters http://www.opencalais.com which takes a piece of text (or a web page) and returns a structured XML file containing the entities found; these include cities, provinces or states, countries, continents and regions. The next step is to convert strings in the XML returned by OpenCalais into geographic locations using the GeoNames service. The steps for this process are essentially the same as for the longer affiliation strings above, although usually with fewer steps as there are generally fewer tokens found in these strings.

In the field of Named Entity Recognition (NER) the question of how to determine which NER system is best is a hard problem [62]. Consider a tagger that decides that every word in your document is a named entity. If the only score we were using was "did it correctly tag all the named entities as named entities?" it would be a winner with 100% accuracy. This is clearly of no use to researchers so the NER community commonly uses 4 scores to keep track of how well a tagger is doing:

• True Positives - Named entity (NE) tagged as NE

• True Negatives - normal words tagged as normal words

• False Positives - normal words tagged as NE

• False Negatives - NE tagged as normal words

Given these 4 scores for a document we can calculate precision as *TP/(TP+FP) *and recall as *TP/(TP+FN)*. So precision shows how many of the named entities that were tagged were actually right while recall shows the proportion of named entities in the document that were actually tagged. Usually as either recall or precision goes up the other goes down. Which measure you should consider more important depends on your application. In some cases, missed entities are most serious and in others false positives are the worse problem. The F-Measure (or F-Score) is the harmonic mean of precision and recall that is an attempt to smooth out the related variation of the two measures. It is defined as *F = (2×PxR)/(P+R)*. In general a higher F-Score is better when you are comparing programs.

We investigated the performance of the following online services in tagging named entities (NE):

• Calais - http://viewer.opencalais.com/

• Metacarta - http://ondemand.metacarta.com/?method=GeoTagger

• Tagthenet - http://www.tagthe.net/

• Alchemy - http://www.alchemyapi.com/api/demo.html

• Zemanta - http://developer.zemanta.com/wiki

The results obtained in a simple test of the NER capabilities of these online services are seen in Table 2. It is quite clear that (at the time these calculations were made and for the kinds of tasks we are focusing on) the OpenCalais service performs much better in terms of precision and F-Measure, while MetaCarta's service is better when recall is considered. In the GeoJunction service, the decision to use the OpenCalais service was made based on the results in Table 2 (that compares five online NER services).

**Table 2:** Comparison of precision, recall and F-measure.

Service	Precision	Recall	F- Measure
Calais	0.99	0.63	0.76
Metacarta	0.52	0.83	0.60
Alchemy	0.75	0.39	0.49
Tagthenet	0.47	0.26	0.31
Zemanta	0.19	0.24	0.20

### For Places - Disambiguate/geolocate

As each place name is extracted from the document, it is returned to a module that attempts to disambiguate it and geolocate the resulting place. Disambiguation involves taking a raw place name like 'London' and determining which of the 2683 places called London http://ws.geonames.org/search?q=london in the GeoNames gazetteer is the correct one. While there is a growing literature discussing methods and algorithms to carry out this process, most assume either that the user has a pre-tagged corpus to train a machine learning method on [63], or that more information than simply London is available to help disambiguate the place name (e.g., a reference to Canada in the document may indicate London, Ontario is correct, while a mention of Columbus might point towards London, Ohio.) [64]. In the current case, with PubMed as the information source, the majority of abstracts are too short to provide this sort of additional information. Thus, external knowledge such as the population size and administrative hierarchy of places is used for first approximation disambiguation based on probabilities.

### For Places - Geocode and encode in spatial database

Once the document has been processed to extract and disambiguate its geographic locations, both those related to where it was written and where it is about, the third step is to retrieve coordinates for (geocode) the places identified. This is a simple database query to the GeoNames database; the system originally made an HTTP request to the GeoNames server and parsed the returned XML file. To optimise the speed of the lookup (and save load on the GeoNames servers) later versions of the code make a database query to a locally stored version of the GeoNames data.

Once coordinates are determined, to allow further analysis of the place names in the document, the places and their locations are then stored in a PostgreSQL-PostGIS database along with any relevant data about the document itself. *HEALTH GeoJunction *includes a single geographically enhanced table to store locations and then a pair of tables that store references (joins) from the articles table to the locations table. This allows spatially aware SQL queries to be made on the source and reference locations of documents (combined with other attributes of the documents as required).

Our current re-engineering of the system is implementing a modular structure. This will make it practical to substitute alternative methods for any of the steps outlined above and to carry out formal tests of method performance.

## Competing interests

The authors declare that they have no competing interests.

## Authors' contributions

AM conceptualized the project, guided design, and took the lead on writing the manuscript. MS contributed to system design, developed and implemented the initial web client interface, and helped to draft the manuscript. IT developed and implemented the server-side computational methods and query mechanism, contributed to overall system design, and helped to draft the manuscript. SP developed and implemented version two of the client interface (presented here), improved system performance, and contributed to the manuscript. All authors read and approved the final manuscript.
